# Analysis of sea-island cotton and upland cotton in response to *Verticillium dahliae* infection by RNA sequencing

**DOI:** 10.1186/1471-2164-14-852

**Published:** 2013-12-05

**Authors:** Quan Sun, Huaizhong Jiang, Xiaoyan Zhu, Weina Wang, Xiaohong He, Yuzhen Shi, Youlu Yuan, Xiongming Du, Yingfan Cai

**Affiliations:** 1 State Key Laboratory of Cotton Biology, Henan Key Laboratory of Plant Stress Biology, School of Life Sciences, Henan University, Kaifeng 475004, China; 2 College of Bioinformation, Chongqing University of Posts and Telecommunications, Chongqing 400065, China; 3 State Key Laboratory of Cotton Biology, Cotton Research Institute of the Chinese Academy of Agricultural Sciences, Anyang, Henan 455000, China

**Keywords:** Cotton, *Verticillium dahliae*, RNA sequencing, Transcriptome analysis, Mechanism of resistance

## Abstract

**Background:**

Cotton Verticillium wilt is a serious soil-borne vascular disease that causes great economic loss each year. However, due to the lack of resistant varieties of upland cotton, the molecular mechanisms of resistance to this disease, especially to the pathogen *Verticillium dahliae*, remain unclear.

**Results:**

We used the RNA-seq method to research the molecular mechanisms of cotton defence responses to different races of *Verticillium dahliae* by comparing infected sea-island cotton and upland cotton. A total of 77,212 unigenes were obtained, and the unigenes were subjected to BLAST searching and annotated using the GO and KO databases. Six sets of digital gene expression data were mapped to the reference transcriptome. The gene expression profiles of cotton infected with *Verticillium dahliae* were compared to those of uninfected cotton; 44 differentially expressed genes were identified. Regarding genes involved in the phenylalanine metabolism pathway, the hydroxycinnamoyl transferase gene (*HCT*) was upregulated in upland cotton whereas *PAL*, *4CL*, *CAD*, *CCoAOMT*, and *COMT* were upregulated in sea-island cotton. Almost no differentially expressed genes in this pathway were identified in sea-island cotton and upland cotton when they were infected with *V. dahliae* V991 and *V. dahliae* D07038, respectively.

**Conclusions:**

Our comprehensive gene expression data at the transcription level will help elucidate the molecular mechanisms of the cotton defence response to *V. dahliae*. By identifying the genes involved in the defence response of each type of cotton to *V. dahliae*, our data not only provide novel molecular information for researchers, but also help accelerate research on genes involved in defences in cotton.

## Background

Cotton is the nature fibre crop with the greatest economic importance. Cotton Verticillium wilt (CVW) is a serious soil-borne vascular disease caused by *Verticillium dahliae*[1]. The disease can cause cotton yellowing, wilt, defoliating, and finally death [2]. CVW was first reported in upland cotton in the United States, and spread to China on imported American cotton in 1935[3-5]. At present, more than 200 million hectares of cotton are subject to CVW, and the economic loss is tremendous every year, especially defoliated CVW [6]. Currently, no fungicides are available to cure commercial upland cotton (*Gossypium hirsutum L.,* the main cultivated cultivar) once they become infected, although the sea-land cotton species (*Gossypium barbadense L.*, not the main cultivated cultivar) is immune to *V. dahliae*. Because of the difficulty obtaining a new highly resistant upland cotton breed by cross-breeding of upland cotton and sea-island cotton, no completely suitable high-resistance upland cotton variety exists. The breeding of disease-resistant cultivars remains the primary control method for cotton. At present, the primary method of breeding for disease-resistant cotton is crossbreeding of resistant cotton, which has been used to create a series of varieties with resistance to CVW [7,8].

In recent years, researchers have investigated the molecular mechanisms of resistance to *V. dahliae* with the aim of ultimately using genetic engineering to breed cultivars resistant to CVW. Resistance to CVW is controlled by a single dominant gene in sea-island cotton, whereas that in upland cotton may be controlled by either a single dominant gene or two dominant genes [5,8-13]. When cotton is infected by the CVW pathogen, the defence system is activated, generating a series of cascade reactions. Three aspects of this system, tissue resistance, physiological and biochemical resistance, and micro-organism resistance, are involved in the mechanism of resistance [14,15]. Several *V. dahliae* resistance-related genes have been isolated and studied, including the following four groups: cinnamyl alcohol dehydrogenase (*CAD*), farnesyl diphosphate synthase, isopentenyl diphosphate isomerase, and 3-hydroxy-3-methylglutaryl coenzyme A reductase, the key regulatory enzymes in the synthesis of phytoalexin, which can kill pathogens through increased local concentrations in the plant [16,17] (Group 1); lipid transfer proteins, chitinase, β-1,3 glucanase, nonexpressor of pathogenesis-related genes 1, thionin, gastrodia antifungal protein, and lectin-like protein, which may play important roles in the response to pathogen infections in cotton plants [18,19] (Group 2); phenylalanine ammonia lyase (*PAL*), cinnamate-4-hydroxylase, peroxisome, polygalacturonase-inhibiting proteins, and laccase, which are defence proteins that can slow down the spread of pathogens [20,21] (Group 3); and resistance gene analogues and defence gene analogues that have been cloned and further transformed by genetic engineering for functional research, such as lipid transfer protein, nonrace-specific disease resistance 1, MAP kinase kinase 2, and others [9,22] (Group 4).

The phenylpropanoid pathway plays a key role in cotton resistance to *V. dahliae*[8,23-25]. Even so, the molecular mechanisms of *V. dahliae* resistance are unclear. In recent years, novel, high-throughput, deep-sequencing transcriptome analysis, termed RNA-seq, has made it possible to efficiently generate large-scale expressed sequence tag (EST) libraries and improved the speed of gene discovery [26]. Although RNA-seq technologies have been used for researching gene expression profiles in the resistance response of sea-island cotton to *V. dahliae*, the phenylpropanoid metabolism pathway and genes associated with lignin metabolism has been found to play a central role [25]. Whether a different response mechanism to the wilt fungus *V. dahliae* exists in cotton of different races and whether different response mechanisms exist in different cotton species (*G. hirsutum* L. and *G. barbadense* L.) are questions for further research. Therefore, in this study, four samples, including ZhongZhiMian KV-1 (*G. hirsutum* L.) and XinHai 15 (*G. barbadense* L.) infected with *V. dahliae* strain V991 (highly toxic) and D07038 (intermediately toxic), respectively, and two uninfected samples include ZhongZhiMian KV-1 (*G. hirsutum* L.) and XinHai 15 (*G. barbadense* L.) were sequenced in order to addressed the above issues using RNA-seq technologies.

## Results

### Illumina sequencing and sequence assembly

**Figure 1:**
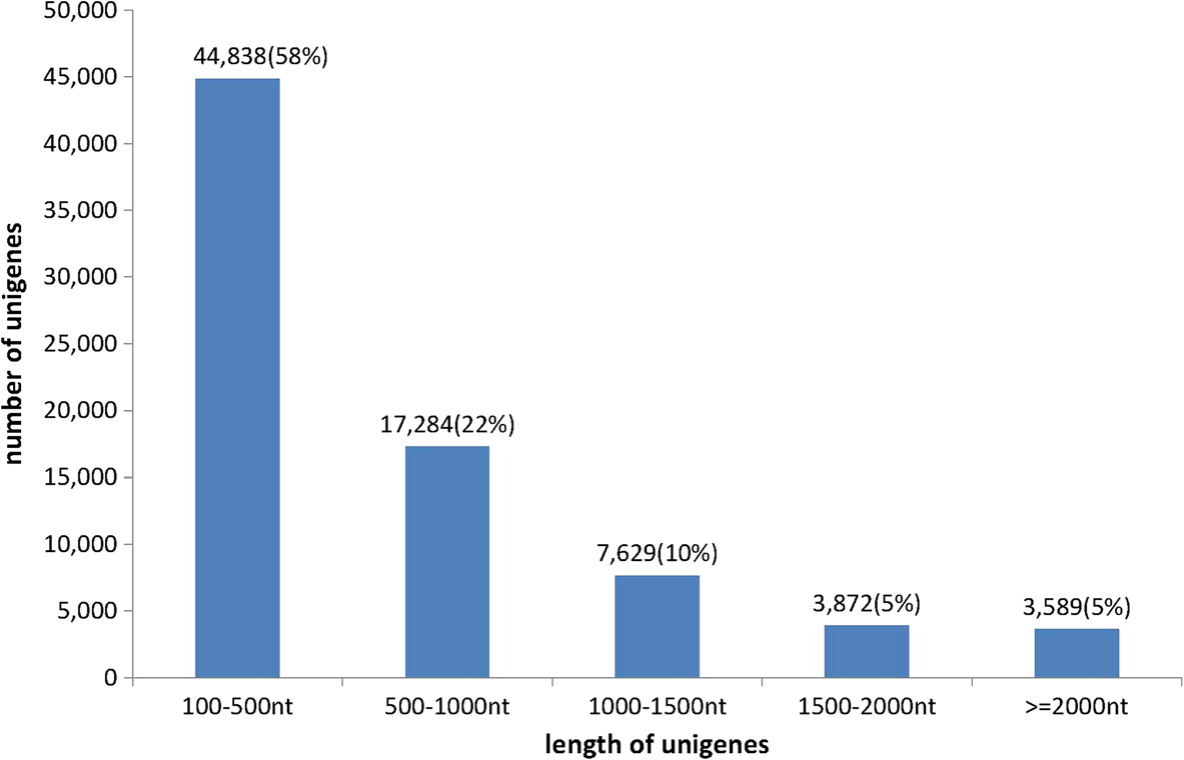
**Unigene size distribution.** Bracket show the ratio of total unigenes.

A total of 56,836,100 reads (accumulated length, 4,652,696,700 bp; SRA accession number SRX128210) were generated through Illumina sequencing and assembled into 167,545 contigs. Then the contigs were further assembled into 77,212 unigenes, with a mean length of 666 bp. The size distribution indicated that 44,838 (58%) unigenes were 100–500 bp, and 42% unigenes were greater than 500 bp (Figure 1). To evaluate the quality of the data set, the ratio of the gap length to the length of the assembled unigenes was analysed. All of the unigenes showed gap lengths of ≤5%.

### Functional annotation and classification

All of the unigenes were compared to the sequences in public databases, including the NCBI nonredundant protein (nr) database, the Clusters of Orthologous Groups (COG) database, the Swiss-Prot protein database, and the KEGG database, using BLASTX with a cutoff e-value of 10^-5^. A total of 66,208 unigenes (78% of all unigenes) returned a significant BLAST result.

**Figure 2:**
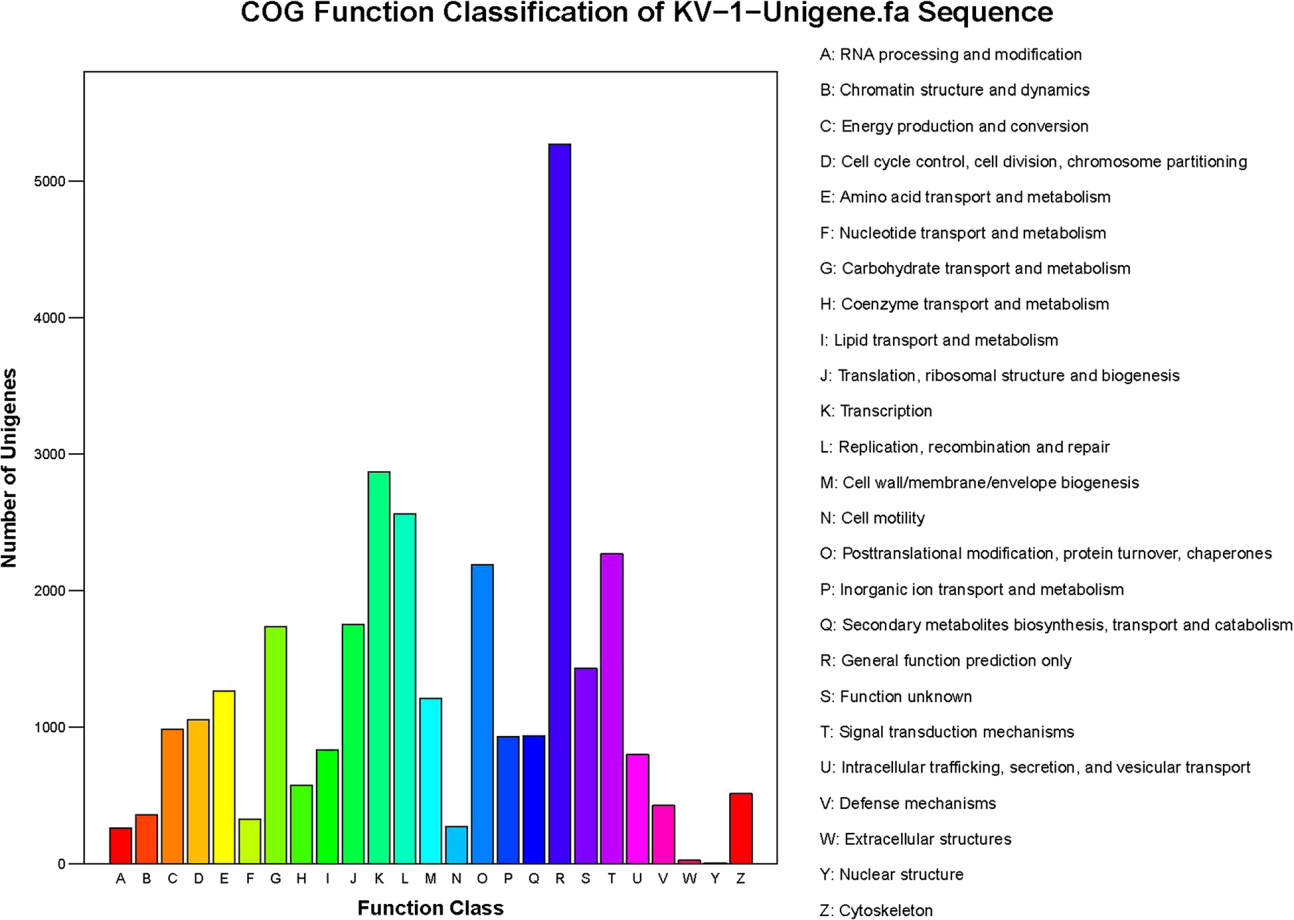
**COG Function Classification of all unigene.** A total of 16,789 unigenes showing significant homology to the COGs database have a COG classification among the 25 cateories.

The assembled unigenes were compared against the COG database to phylogenetically analyse widespread domain families. The results revealed 16,789 unigenes with significant homology and assigned them to the appropriate COG clusters. These COG classifications were grouped into 25 functional categories (Figure 2). Among these COG categories, the cluster 'general function’ (5274; 31.4%) represented the largest group, followed by 'transcription’ (2869; 17.1%); 'replication, recombination, and repair’ (2561; 15.3%); 'signal transduction mechanisms’ (2268;13.5%); 'posttranslational modification, protein turnover, chaperones’ (2192; 13.1%); 'translation, ribosomal structure, and biogenesis’ (1752; 10.4%); and 'carbohydrate transport and metabolism’ (1736; 10.3%).

**Figure 3:**
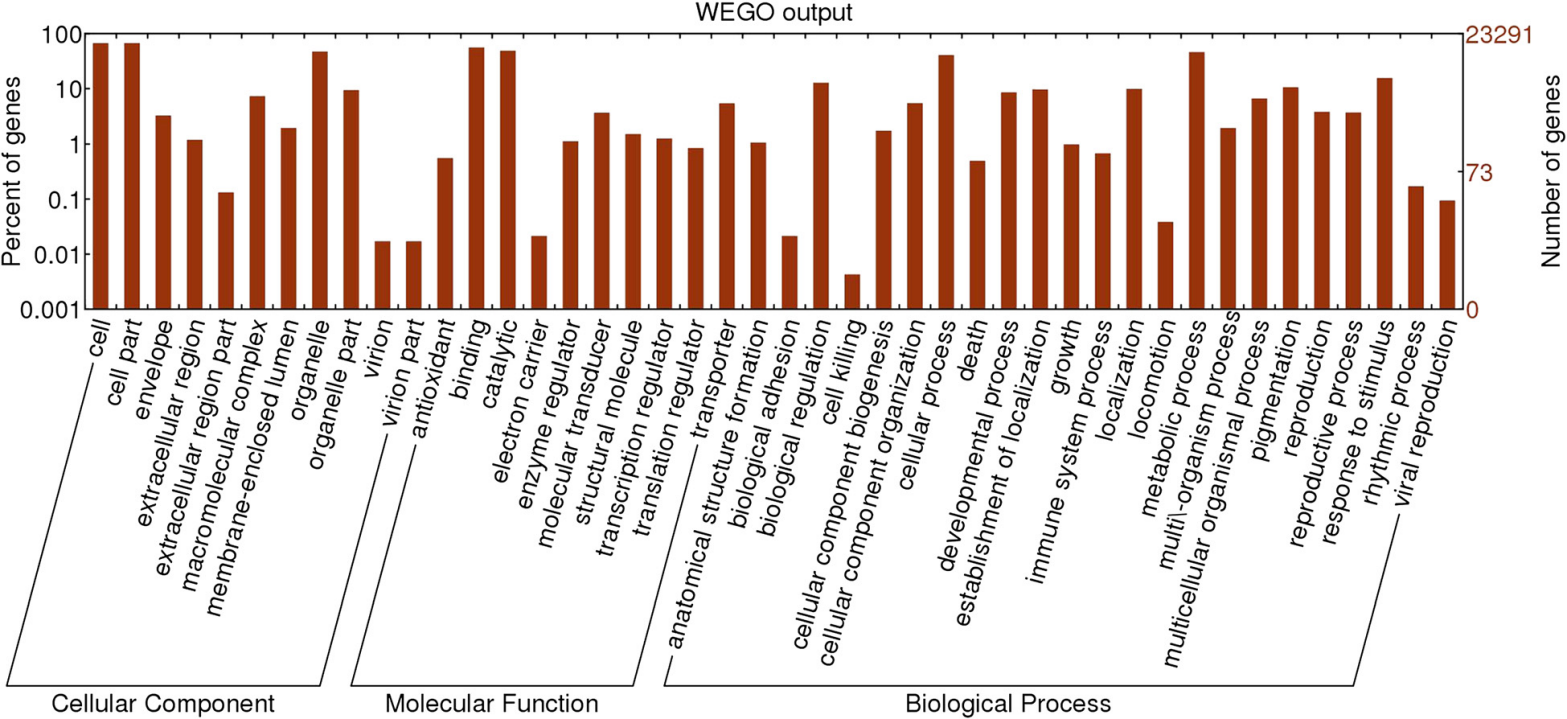
Gene ontology classification of the cotton transcriptome.

Gene Ontology (GO) assignments were used to classify the functions of the predicted cotton genes. Based on sequence homology, 23,291 sequences were categorised into 44 functional groups (Figure 3). In each of the three main categories (biological process, cellular component, and molecular function) of the GO classification, the major subcategories were as follows: six subcategories for biological process ('biological regulation’ , 'cellular process’ , 'localization’ , 'metabolic process’ , 'regulation of biological process’ , and 'response to stimulus’); three sub-categories for cellular components ('cell’ , 'cell part’ and 'organelle’); and two sub-categories for molecular function ('binding’ and 'catalytic activity’). Only a few genes were clustered in terms of 'biological adhesion’ , 'cell killing’ , 'locomotion’ , 'pigmentation’ , 'rhythmic process’ , 'viral reproduction’ , 'cell junction’ , 'extracellular region’ , 'virion’ , and 'translation regulator activity’.

Functional classification and pathway assignment were performed using the KEGG database [27]. In total, 28,395 unigenes were assigned to 126 KEGG pathways (see Table 1). The major pathways were 'metabolic pathways’ , 'biosynthesis of secondary metabolites’ , 'plant hormone signal transduction’ , and 'plant-pathogen interaction’; the gene numbers and percentages assigned to these pathways were 5912 (20.82%), 2959 (10.42%), 2243 (7.9%), and 2238 (7.88%), respectively.

**Table 1:** Pathway annotation to KEGG

**ID**	**Pathway**	**No. of unigenes (28395)**
1	Metabolic pathways	5912 (20.82%)
2	Biosynthesis of secondary metabolites	2959 (10.42%)
3	Plant hormone signal transduction	2243 (7.9%)
4	Plant-pathogen interaction	2238 (7.88%)
5	RNA transport	1102 (3.88%)
6	Spliceosome	915 (3.22%)
7	Ribosome biogenesis in eukaryotes	907 (3.19%)
8	RNA degradation	818 (2.88%)
9	Protein processing in endoplasmic reticulum	791 (2.79%)
10	Purine metabolism	732 (2.58%)
11	Endocytosis	715 (2.52%)
12	Glycerophospholipid metabolism	666 (2.35%)
13	mRNA surveillance pathway	654 (2.3%)
14	Starch and sucrose metabolism	596 (2.1%)
15	Ribosome	574 (2.02%)
16	Ubiquitin mediated proteolysis	512 (1.8%)
17	Phenylpropanoid biosynthesis	467 (1.64%)
18	Ether lipid metabolism	455 (1.6%)
19	Pyrimidine metabolism	387 (1.36%)
20	Circadian rhythm - plant	384 (1.35%)
21	Amino sugar and nucleotide sugar metabolism	359 (1.26%)
22	Phagosome	357 (1.26%)
23	Peroxisome	355 (1.25%)
24	Stilbenoid, diarylheptanoid and gingerol biosynthesis	346 (1.22%)
25	Flavonoid biosynthesis	342 (1.2%)
26	Oxidative phosphorylation	341 (1.2%)
27	Glycolysis/Gluconeogenesis	320 (1.13%)
28	Limonene and pinene degradation	301 (1.06%)
29	Phosphatidylinositol signaling system	290 (1.02%)

Note: Data only showed the annotation unigenes percent of each pathway more than 1%.

### DGE library sequencing

Six cotton digital gene expression (DGE) profiling libraries were sequenced (1 ~ 6, corresponding to the SRA accession numbers SRX128211, SRX128213, SRX128212, SRX128214, SRX128215, and SRX128216, respectively), which generated approximately 11 to 12 million high-quality reads for each library (Table 2). The percentage of clean reads among the raw reads in each library was >99% (Table 3). Among the clean reads, the number of sequences that could be mapped to unigenes ranged from 9.4 to 10.2 million, and the percentage of clean reads was above 79% in the six libraries. As Table 2 shows, the vast majority of these mapped reads were uniquely matched to unigenes (> 55%), and the percentage of multi-position matched reads was 24%.

**Table 2:** Statistics of DGE sequencing

**Total reads**	**12230124(100%)**	**11875055(100%)**	**11764630(100%)**	**12511454(100%)**	**12167879(100%)**	**12420918(100%)**
Total mapped reads	9770169(79.89 %)	9493222(79.94 %)	9411269(80 %)	9969428(79.68 %)	9867697(81.1 %)	10150382(81.72 %)
Perfect match	6659597(54.45 %)	6469378(54.48 %)	6478583(55.07 %)	6778488(54.18 %)	6721125(55.24 %)	6954849(55.99 %)
<=2 bp mismatch	3110572(25.43 %)	3023844(25.46 %)	2932686(24.93 %)	3190940(25.5 %)	3146572(25.86 %)	3195533(25.73 %)
Unique match	6853979(56.04 %)	6623521(55.78 %)	6692373(56.89 %)	7004728(55.99 %)	7165038(58.88 %)	7483020(60.25 %)
Multi-position match	2916190(23.84 %)	2869701(24.17 %)	2718896(23.11 %)	2964700(23.7 %)	2702659(22.21 %)	2667362(21.47 %)
Total unmapped reads	2459955(20.11 %)	2381833(20.06 %)	2353361(20 %)	2542026(20.32 %)	2300182(18.9 %)	2270536(18.28 %)

Note: 1: KV1/D; 2: XH/D; 3: ZKV1/V; 4:XH/V; 5:KV1/U; and 6: XH/U.

**Table 3:** Different components of the raw reads in each sample

**Summary**	**Clean reads only**	**Only adaptor**	**Containing N**	**Low quality**
1	12230124(99.39%)	54281(0.44%)	788(0.01%)	19459(0.16%)
2	11875055(99.45%)	45599(0.38%)	0	19899(0.17%)
3	11764630(99.38%)	53254,0.45%)	745(0.01%)	19561(0.17%)
4	12511454(99.42%)	51023(0.41%)	0	21851(0.17%)
5	12167879(99.42%)	49895(0.41%)	749(0.01%)	20211(0.17%)
6	12420918(99.48%)	49511,0.40%)	1094(0.01%)	14766(0.12%)

Note: 1: KV1/D; 2: XH/D; 3: ZKV1/V; 4:XH/V; 5:KV1/U; and 6: XH/U.

### Gene expression variation among the different samples

**Figure 4:**
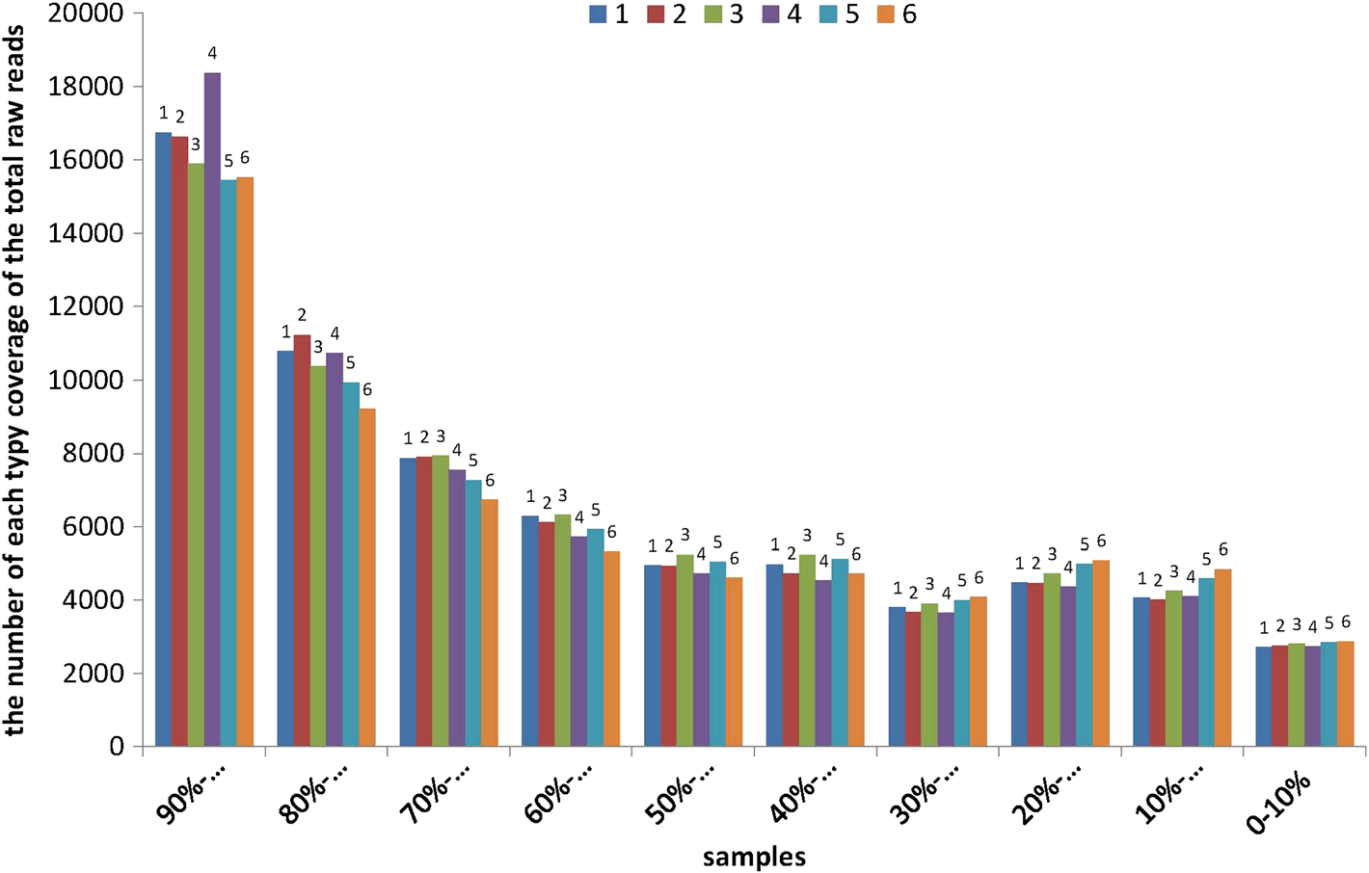
**Distribution of reference unigenes’ coverage in each sample.** 1: KV1/D; 2: XH/D; 3: ZKV1/V; 4:XH/V; 5:KV1/U; and 6: XH/U.

First, to evaluate the DGE data, we analysed the distribution of unigene coverage in each sample, which is the number of clean reads that aligned to the reference unigenes. As shown in Figure 4, most unigene coverage was >50% (1, 69% of all unigenes; 2, 70% of all unigenes; 3, 69% of all unigenes; 4, 78% of all unigenes; 5, 67% of all unigenes; and 6, 66% of all unigenes). Second, the number of clean reads was calculated and the gene expression level was calculated using the reads per kb per million reads method for each unigene, and then the differentially expressed genes (DEGs) were identified in different samples.

**Figure 5:**
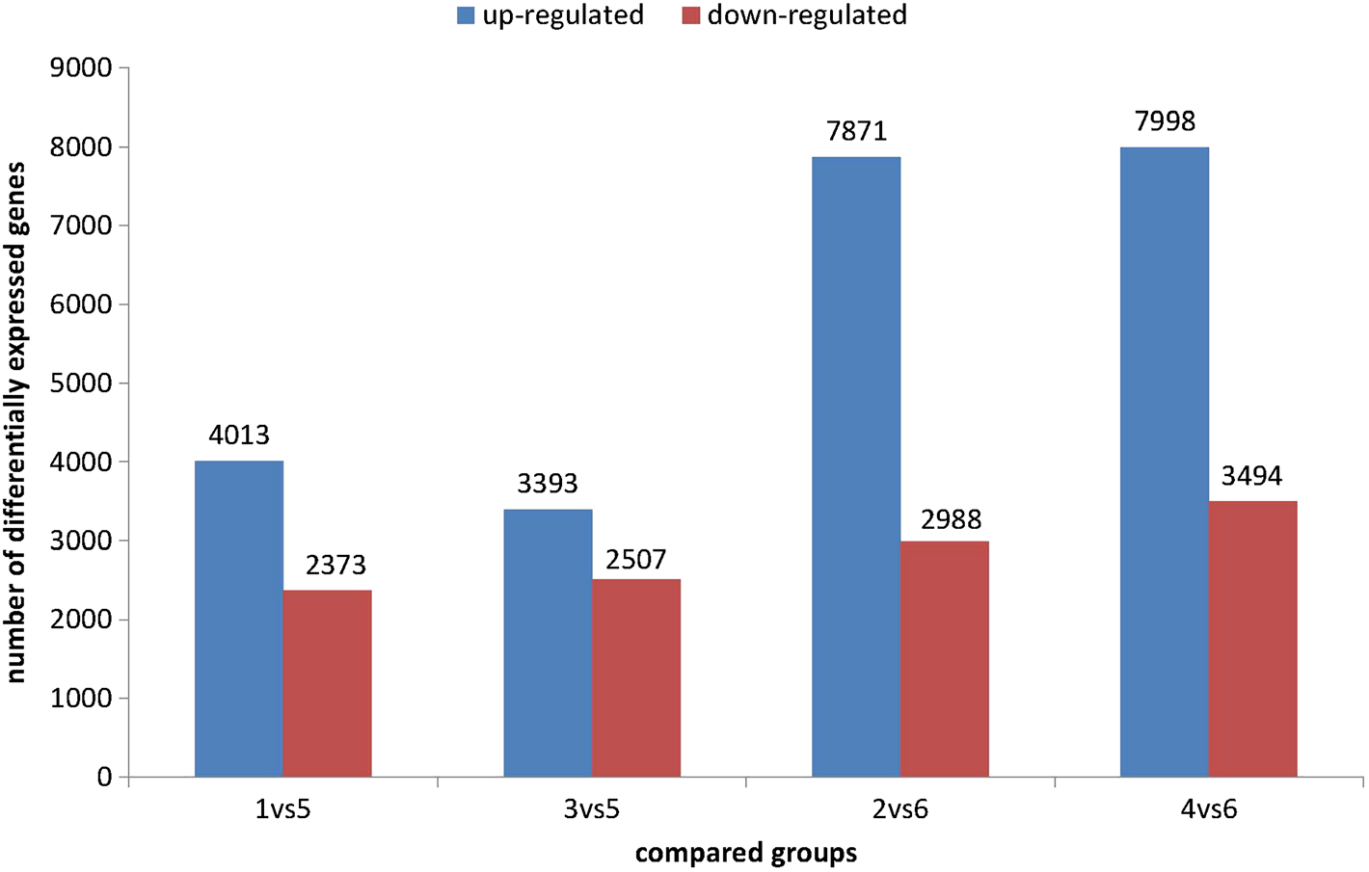
**Numbers of differnet expressed unigenes in each comparison.** The numbers on column showed quantity of up-regulated (blue) and down-regulated (red) unigenes. 1: KV1/D; 2: XH/D; 3: ZKV1/V; 4:XH/V; 5:KV1/U; and 6: XH/U.

Variation in gene expression was identified based on comparisons of the above. The following significant DEGs were identified: (a) between samples 1 (KV1/D) and 5 (KV1/U), 4013 and 2373 genes were up- and downregulated, respectively; (b) between samples 3 (KV1/V) and 5 (KV1/U), 3393 and 2507 genes were up- and downregulated, respectively; (c) between samples 2 (XH/D) and 6 (XH/U), 7871 and 2988 genes were up- and downregulated, respectively; and (d) between samples 4 (XH/V) and 6 (XH/U), 7998 and 3494 genes were up- and downregulated, respectively (Figure 5).

### Genes related to resistance to CVW resistance

Based on the above comparisons, the DEGs were functionally analysed using MapMan software. The results showed that a total of 1975 genes occurred simultaneously in the four comparisons above (a–d), including 1535 upregulated genes and 440 downregulated genes. These genes were principally associated with cell wall, lipids, and secondary metabolism and so on (Additional file 1). To identify cotton genes most associated with resistance to CVW, an absolute value of the fold change times > =5 was used as a threshold, resulting in 44 DEGs being screened out (including 41 upregulated and 3 downregulated genes, Table 4).

### Validation of RNA-Seq-based gene expression

**Figure 6:**
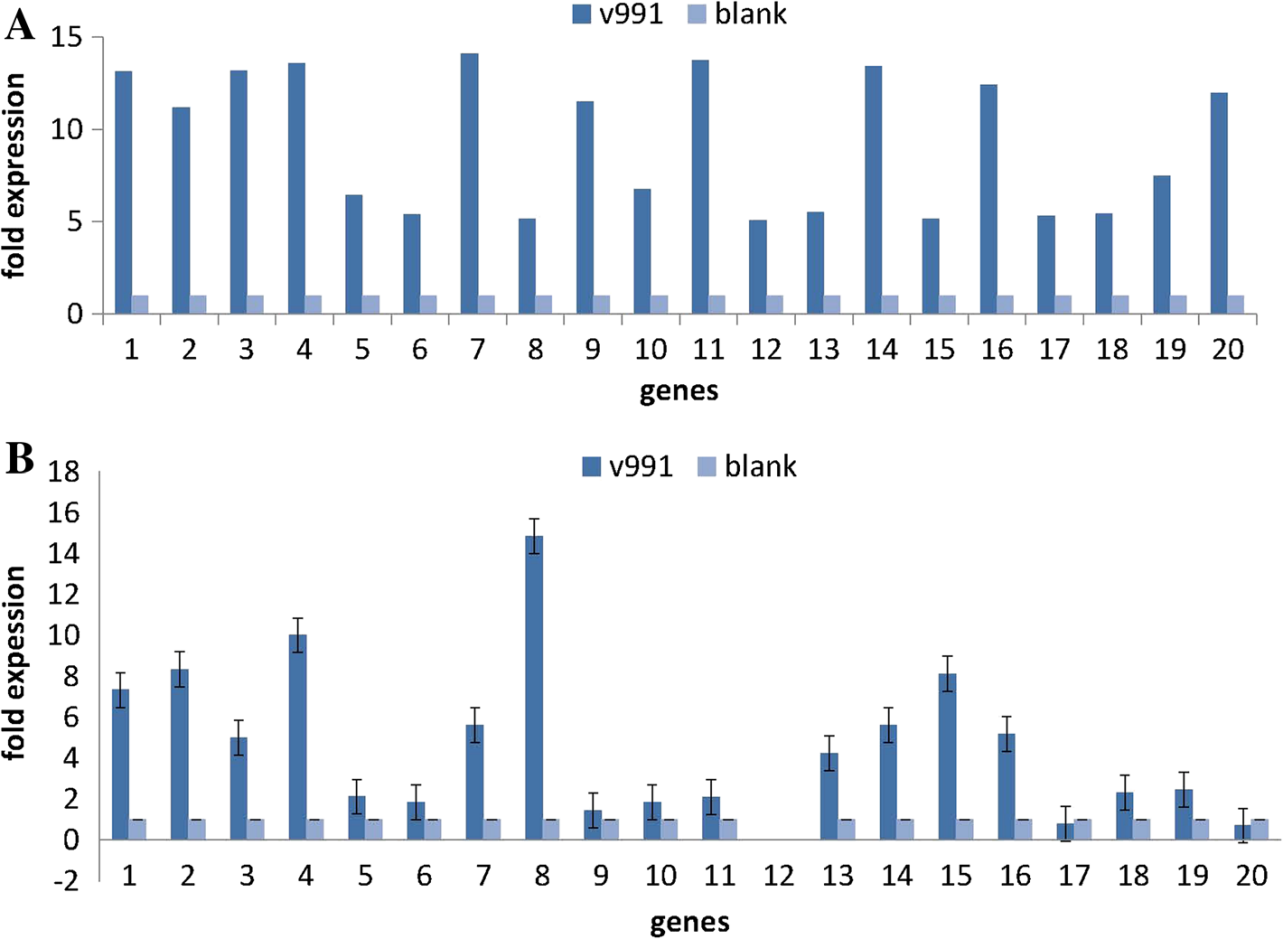
**Expression pattern of the selected genes in G.hirsutum. (A)** Gene expression data for DGE analysis. The fold changes of the genes were shown on the y-axis. **(B)** The qRT-PCR analysis of gene expression data.V991 mean ZhongZhiMian KV-1 infected by V991, blank show ZhongZhiMian KV-1 uninfected. Error bars represent SE for three independent experiments.

To validate the assembled sequences and the expression profiles obtained by RNA-Seq, real-time RT-PCR was performed on 20 of the above DEGs. Gene expression levels in V991 and the controls were compared using qRT-PCR. Of the 20 genes, all but gene 12 (id: unigene 64452) were detected. For the 19 detected genes, the upregulation trend obtained in real-time RT-PCR expression was in agreement with the RNA-Seq data, except for genes 17 and 20 (unigene 51427 and unigene 12430). However, for most of the genes, the upregulated range in real-time RT-PCR was lower than in the DGE data (Figure 6).

## Discussion

Cotton is an economically important crop, and cotton CVW causes significant economic losses. Although some cotton cultivars resistant to *V. dahliae* have been generated using conventional breeding techniques, the molecular mechanisms of disease resistance in cotton are still unclear. Xu et al. [25] found that lignin metabolism is a key pathway in the resistance of cotton, but the authors mainly analysed the RNA-seq results by analysing the resistance mechanism of the resistant *Gossypium barbadense* in contrast to the susceptibl*e Gossypium hirsutum*. Are other key genes involved in the resistance of cotton? In this study, we used the commercial upland cotton (*Gossypium hirsutum*) variety ZhongZhiMian KV-1 and the sea-island cotton Xinhai 15, both highly resistant to *V. dahliae*, to study the resistance relationships between different cotton species and different *V. dahliae* strains.

Using transcriptome sequence analysis, 56,836,100 reads were obtained, corresponding to about 4.7 Gb of raw sequence data. Then the raw sequences were further assembled using the short-reads assembly program Trinity and used to predict unigenes. In spite of the genome sequence of a diploid cotton *Gossypium raimondi* had been published, whose progenitor is the putative contributor of the D-subgenome to cotton species *Gossypium barbadense* and *Gossypium hirsutum*[28,29], due to the lack of A-genome sequence information, the D-genome sequence were not used in our bioinformation analysis in this study, and the quality of these assembled unigenes was confirmed by qRT-PCR. The results showed that signals for 19 out of 20 tested genes were detected, demonstrating the high quality of the assembled results, and also indicating that relatively short reads from Illumina sequencing can be effectively assembled and used for novel gene discovery. The 66208 predicted unigenes were subjected to BLAST annotation; 78% of the unigenes returned a significant BLAST result. About 20% of the predicted unigenes could not be assigned annotation information, which may be caused by the limited information about the genomes or transcriptomes of cotton and its related species. Some unigenes were too short to allow statistically meaningful matches. From the KEGG annotation results, 2238 unigenes (7.88% of all KEGG annotation unigenes) were found to be involved in the group 'plant-pathogen interaction’, which may be associated with the defence response of cotton to *V. dahliae*. This group was the fourth largest group in our results, which suggests that it is feasible through the immense capacity of Illumina sequencing to discover genes related to cotton resistance to *V. dahliae* in metabolic pathways.

To understand the regulatory pathways and molecular mechanisms of resistance to *V. dahliae*, we created six DGE libraries to analyse gene expression patterns following inoculation with *V. dahliae*. About 80% of the reads were mapped to our transcriptome reference database. The high proportion of mapped reads supports the validity of our analysis. The quality of the DGE libraries was also confirmed by qRT-PCR analysis. Because the cotton disease resistance-related genes may begin to be expressed after inoculation with *V. dahlia*, cotton seedlings of different species inoculated with different races of *V. dahliae* were compared to controls. Different genes may be involved in disease resistance, and the common genes in the four comparison groups reduced the number of DEGs that may be related to cotton disease resistance. There were no replicated data using the Audic-Claverie method in DGE data analysing, but the results are credible because we applied a series of correction method, such as: a false discovery rate of <0.001 and an absolute value of the log 2 ratio >1 as the threshold were used for judging the significance of the gene expression differences, and combined with the quality results of the DGE libraries been further confirmed by qRT-PCR.

The inoculated *V. dahliae* cotton samples compared with uninoculated samples exhibited much more upregulated genes and downregulated genes, this possibly indicates that *V. dahliae* activates cotton resistance genes during the infection process. Furthermore, the number of upregulated and downregulated genes of *G. barbadense* was much greater than that in *G. hirsutum* (both inoculated with *V. dahliae* races V991 and D07038) in the four compared groups, suggesting that the resistance genes to *V.dahliae* of the sea-island cotton *G. barbadense* is more than that of the upland cotton *G. hirsutum*.

The genes that were upregulated or downregulated more than five fold in the four compared groups are listed in Table 4. It is feasible that the functional genes play key roles in the resistance of cotton to *V. dahliae*. Of these genes, the gene unigene5503_kv-1 was described as a cellulose synthase-like D gene; cellulose synthase-like genes mediate the synthesis of cell wall (1,3;1,4)-beta-D-glucans and/or are required for cellulose deposition [30-33]. Vega-Sánchez et al. found a cellulose synthase-like protein related to defence responses of rice plants [33]. In our study, the cellulose synthase-like D protein was differentially expressed upon infection of cotton seedlings with *V. dahliae*. The genes unigene68082_kv-1 and unigene67909_kv-1 were annotated as encoding xyloglucan endotraglucosylase/hydrolase and xyloglucan 6-xylosyltransferase, respectively. As reported previously, xyloglucan 6-xylosyltransferase can catalyse the formation of UDP-D-xylose-D-glucose from UDP-D-xylose in xyloglucan, and xyloglucan endotransglycosylase/hydrolase can degrade xyloglucan [34-37]. Xyloglucans play an important role in cross-linking adjacent cellulose microfibrils to form a cellulose-xyloglucan network that constitutes the major load-bearing structure of the primary cell wall. Xu et al. found that lignin has a key role in the cotton defence response, suggesting that genes related to cotton cell wall components are involved in the defence response to *V. dahlia*[25]. The gene unigene52341_kv-1 is similar to the terpene synthase 3 gene from *Populus trichocarpa*. Danner et al. found that terpene synthase 3 can activate both mono- and sesquiterpene synthase in *Populus trichocarpa*[38], and in cotton, sesquiterpene phytoalexins are elicited in response to bacterial or fungal infection [39,40]. In conclusion, the four genes mentioned above are probably related to cotton resistance. Three of the genes are related to cell-wall compounds and one gene is related to sesquiterpene metabolism, suggesting that the cell wall and sesquiterpene are involved in the resistance of cotton to CVW. These genes are targets of further research in our next study.

**Table 4:** Different expression genes screening out

**BinCode**	**BinName**	**ID**	**Description**	**1v5**	**2v6**	**4v6**	**3v5**
10.2.1	cell wall.cellulose synthesis.cellulose synthase	unigene5503_kv-1	cellulose synthase-like D5	13.21	14.26	13.38	13.14
10.7	cell wall.modification	unigene68082_kv-1	xyloglucosyl transferase	13.07	14.76	14.18	11.22
16.1.5	secondary metabolism.isoprenoids.terpenoids	unigene52341_kv-1	TPS14 (TERPENE SYNTHASE 14)	11.83	13.03	14.84	13.19
17.5.1	hormone metabolism.ethylene.synthesis-degradation	unigene5647_kv-1	oxidoreductase	13.82	12.63	12.65	13.61
20.2.3	stress.abiotic.drought/salt	unigene23926_kv-1	early-responsive to dehydration protein-related	6.41	6.27	6.15	6.44
20.2.3	stress.abiotic.drought/salt	unigene23927_kv-1	early-responsive to dehydration protein-related	5.25	5.98	5.96	5.39
26.2	misc.UDP glucosyl and glucoronyl transferases	unigene67909_kv-1	XT1 (XYLOSYLTRANSFERASE 1)	13.73	14.14	14.56	14.13
26.1	misc.cytochrome P450	unigene25666_kv-1	fatty acid (omega-1)-hydroxylase/ oxygen binding	5.03	9.4	8.89	5.15
26.28	misc.GDSL-motif lipase	unigene5707_kv-1	LTL1 (LI-TOLERANT LIPASE 1)	11.21	13.81	12.54	11.52
27.3.3	RNA.regulation of transcription.AP2/EREBP, APETALA2/Ethylene-responsive element binding protein family	unigene11425_kv-1	ABR1 (ABA REPRESSOR1)	5.06	8.78	8.51	6.77
27.3.3	RNA.regulation of transcription.AP2/EREBP, APETALA2/Ethylene-responsive element binding protein family	unigene13946_kv-1	ap2/EREBP transcription factor	13.34	13.56	14.29	13.77
27.3.6	RNA.regulation of transcription.bHLH,Basic Helix-Loop-Helix family	unigene64452_kv-1	basic helix-loop-helix (bHLH) family protein	6.23	15.4	13.06	5.08
27.3.7	RNA.regulation of transcription.C2C2(Zn) CO-like, Constans-like zinc finger family	unigene56521_kv-1	JAZ10 (JASMONATE-ZIM-DOMAIN PROTEIN 10)	6.13	7.61	7.35	5.54
27.3.26	RNA.regulation of transcription.MYB-related transcription factor family	unigene54286_kv-1	ATRL5 (ARABIDOPSIS RAD-LIKE 5)	14.31	12.55	12.98	13.43
28.1.3	DNA.synthesis/chromatin structure.histone	unigene16272_kv-1		12.43	14.56	14.31	12.18
29.4	protein.postranslational modification	unigene9429_kv-1	MAPKKK17	5.27	6.13	5.81	5.18
29.5.11.4.3.2	protein.degradation.ubiquitin.E3.SCF.FBOX	unigene4485_kv-1		14.03	14.2	12.62	12.41
30.3	signalling.calcium	unigene51427_kv-1	calmodulin-binding family protein	6.02	15.52	12.88	5.35
33.99	development.unspecified	unigene25035_kv-1	NAC domain-containing protein 68	5.54	14.28	13.09	5.46
34.3	transport.amino acids	unigene60928_kv-1	amino acid/polyamine transporter II	7.1	7.05	5.87	7.51
34.18	transport.unspecified anions	unigene12430_kv-1	BOR4 (REQUIRES HIGH BORON 4)	10.18	5.7	5.05	11.99

Note: column 5–8 showed the fold change times in four compared groups. 1: KV1/D; 2: XH/D; 3: ZKV1/V; 4:XH/V; 5:KV1/U; and 6: XH/U. The 'not assigned.unknown’ genes were not showed.

**Figure 7:**
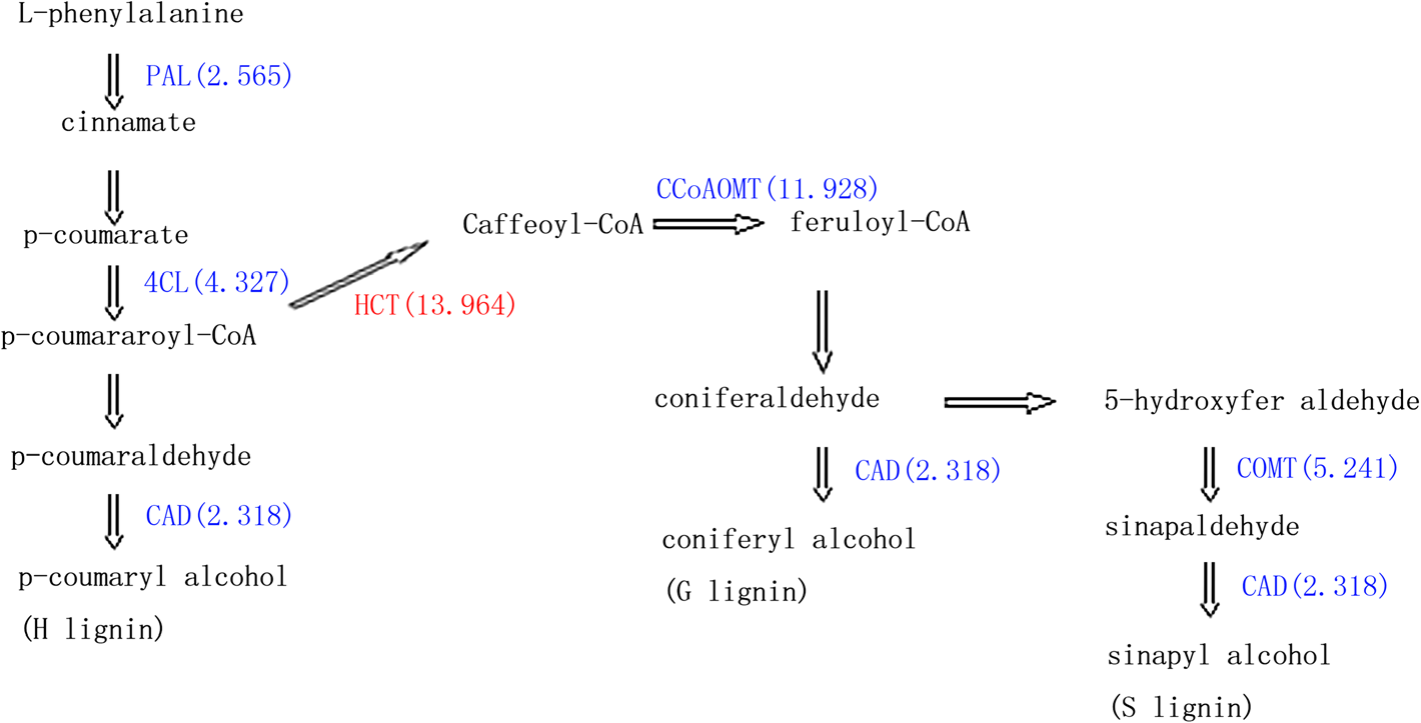
**The unigenes involved in the phenylpropanoid pathway.** Red show up-regulated in compared group of infected and uninfected in upland cotton *G.hirsutum*. Blue display up-regulated in compared group of infected and uninfected in sea island cotton *G. barbadense.* Digital in bracket mean the one biggest fold change times of annotating unigenes. hydroxycinnamoyl transferase (*HC*T), phenylalanine ammonia-lyase (*PA*L), 4-coumarate (*4CL*), cinnamyl alcohol dehydrogenase (*CAD*), caffeoyl-CoA O-methyltransgerase (*CCoAOMT*), caffeoyl O-methyltransgerase (*COMT*).

Other reports have shown that the phenylalanine metabolism pathway is related to the cotton defence response to *V. dahliae*. Therefore, in this study, we also analysed this pathway and identified some DEGs. Different cotton species and races of CVW were used in our study. The DEGs were nearly the same for sea-island cotton and upland cotton infected with different races of *V. dahliae*, but very different when comparing the two groups of sea-island cotton and the two groups of upland cotton. As shown in Figure 7, only one gene, hydroxycinnamoyl transferase (*HCT*), was upregulated and showed a strong change in expression when upland cotton was infected by V. dahliae (V991 or D07038), but five other genes (phenylalanine ammonia-lyase [*PAL*], 4-coumarate [*4CL*], cinnamyl alcohol dehydrogenase [*CAD*], caffeoyl-CoA O-methyltransgerase [*CCoAOMT*], and caffeoyl O-methyltransgerase [*COMT*]) were upregulated and obviously changed in sea-island cotton infected by V. dahliae (including V991 and D07038). As the reports, *HCT* can affect lignification in alfalfa, and lignin plays a key role in the cotton defence response, so the same function may exit in upland cotton [41].The results suggest that *HCT* is related to the upland cotton defence response to V. dahlia, but has little role in the sea-island cotton defence response, and the other five genes (especially the most upregulated gene, *CCoAOMT*) may be related to the defence response of sea-island cotton but not that of upland cotton. In other words, different disease-resistance mechanisms may exist in sea-island cotton and upland cotton. This may be the reason that the genes *HCT* and *CCoAOMT* were not identified.

## Conclusions

Although the molecular functions of the cotton defence response to *V. dahliae* and the associated signal transduction pathways remain largely unknown, the present transcriptome analysis provides valuable information regarding cotton resistance, which may facilitate future investigations of the detailed regulatory mechanisms and pathways. Using Illumina sequencing technology, we obtained about 5 Gb of raw sequence data and predicted 77,212 assembled unigenes, 66,208 of which were able to be annotated. Finally 44 differentially expressed genes were identified. The results suggest that HCT in upland cotton and *CCoAOMT*, *PAL*, *4CL*, *CAD*, and *COMT* in sea-island cotton are involved in cotton defence response to *V. dahliae*. We believe that our data not only provide novel molecular information, but will also help to accelerate research into gene functions of cotton defence responses.

## Methods

### Preparation of material

One highly aggressive strain of the defoliating fungus *V. dahliae*, V991, and one intermediately aggressive strain, D07038, from the Institute of Plant Protection (IPPCAAS) and the Cotton Institute of the Chinese Academy of Agriculture Sciences(CICAAS), respectively, were used for inoculation [2]. The cotton varieties ZhongZhiMian KV-1 (*G. hirsutum* L., from IPPCAAS) and XinHai 15 (*G. barbadense* L.,from the Economic Crop Institute of the Xinjiang Academy of Agriculture Sciences,ECIXAAS, and CICAAS) resistant to *V. dahliae* were grown in sterilized soil (a mix of peat and sawdust) in a natural environment [42]. Each seedling was inoculated with 10 mL *V. dahliae* spore suspension of 2 × 10^7^ spores per mL by watering injured roots from plants at the two-true-leaf growth stage. Control plants were not inoculated but were otherwise treated and sampled with distilled water in the same way. Plants from six individual seedlings were collected for each treatment at each sampling time point (including 24, 48, and 96 h after inoculation) after washed by 75% alcohol and sterile water.

### cDNA preparation for Illumina sequencing

Total RNA was extracted using a Plant RNA EASYspin Plus Kit (aidlab, Peking, China) according to the manufacturer’s instructions. Six RNA samples (for each sample, all time points of each individual treatment were mixed), including four samples of ZhongZhiMian KV-1 (*G. hirsutum*) and XinHai 15 (*G. barbadense*) infected with *V. dahliae* strain V991 (highly toxic) and D07038 (intermediately toxic), respectively. Two plant samples treated with sterile water were further verified for the quantity of RNA using an ultraviolet spectrometer and electrophoresis on a denaturing formaldehyde agarose gel. The samples were as follows: 1, ZhongZhiMian KV-1 infected with D07038 (named: KV1/D); 2, XinHai 15 infected with D07038(named: XH/D); 3, ZhongZhiMian KV-1 infected with V991(named: KV1/V); 4, XinHai 15 infected with V991(named: XH/V); 5, uninfected ZhongZhiMian KV-1(named: KV1/U); and 6, uninfected XinHai 15(named: XH/U). All samples were mixed equally for transcriptome sequencing, but subjected individually to DGE sequencing. Oligo(dT) beads were used to isolate poly(A) + mRNA from total RNA. Fragmentation buffer was added to disrupt the mRNA into short fragments. These short fragments were used as templates with random hexamer primer to synthesise first-strand cDNA. Second-strand cDNA was synthesised by adding buffer, dNTPs, RNase, and DNA polymerase I. The resulting short fragments were purified with a QiaQuick PCR extraction kit and resolved with EB buffer for end reparation and addition of a poly(A) tail. Next, the short fragments were connected with sequencing adapters. Following agarose gel electrophoresis, suitable fragments were selected as templates for PCR. Finally, the library was sequenced using an Illumina HiSeq™ 2000.

### Analysis of transcriptome sequencing data

The raw data from the images were collected using Illumina GA Pipeline 1.6 by removing low-quality reads (reads with unknown sequences 'N’), adaptor sequence fragments, and empty reads. Next, *de novo* assembly of the transcriptome into unigenes was carried out with Trinity, a short-read assembly program [43]. In brief, Trinity firstly combines reads with certain length of overlap to form longer fragments, which are called contigs. Then the reads are mapped back to contigs; with paired-end reads it is able to detect contigs from the same transcript as well as the distances between these contigs. Finally, Trinity connects the contigs, and get sequences that cannot be extended on either end. Such sequences are defined as Unigenes. Subsequently, a Basic Local Alignment Search Tool (BLAST) BLASTx alignment (e-value < 0.00001) was performed for the unigenes with protein databases, including the non-redundant (nr), Swiss-Prot, Kyoto Encyclopaedia of Genes and Genomes (KEGG), and COG databases, and the best alignments were used to decide the sequence direction of the unigenes. If the results from the different databases conflicted with each other, a priority order of nr, Swiss-Prot, KEGG, and COG was followed to decide the sequence direction of the unigenes. When a unigene was not aligned in any of the above databases, ESTScan was used to predict its coding regions and decide its sequence direction [44]. In the final step, using nr annotation, the Blast2GO program was used to obtain the Gene Ontology (GO) and KEGG annotations for the unigenes [45]. After the GO annotation was obtained for each unigene, WEGO software was used to classify the unigenes by function and to determine the distribution of gene functions in the species at the macro level [46]. All raw transcriptome data have been deposited in SRA (NCBI).

### Analysis and mapping of DGE reads

The raw image data were transformed by base calling into sequence data. To map the DGE reads, the sequenced raw data were filtered to remove adaptor sequences, low-quality sequences (reads with unknown sequences 'N’), empty reads. For reads annotation, clean reads sequences were mapped to our transcriptome reference database, allowing no more than two nucleotide mismatch [43,47]. Gene coverage is the percentage of a gene covered by reads. This value is equal to the ratio of the base number in a gene covered by unique mapping reads to the total bases number of that gene. For gene expression analysis, the number of expressed sequence was calculated and normalised to the number of Reads per Kb per Million reads (RPKM).

### Identification of differentially expressed genes

To compare the differences in gene expression, the reads frequency in each DGE library was statistically analysed according to the method of Audic and Claverie [48]. We used a false discovery rate of <0.001 and an absolute value of the log 2 ratio >1 as the threshold for judging the significance of the gene expression differences [49]. To determine the genes related to disease resistance, the inoculated *V. dahliae* cotton samples (samples 1 to 4) were compared to water-inoculated cotton samples (samples 5 and 6). Next, the differentially expressed genes were subjected to GO and KEGG Ontology (KO) enrichment analysis [27]. At the same time, the differentially expressed genes sequences of each group were uploaded to the Mercator web application to assign MapMan bins, and then differentially expressed genes between the four compared groups of related pathways were screened and analysed using MapMan software [50].

### Data validation by qPCR

Total RNA was extracted as described for DGE library preparation and sequencing. Total RNA (1 μg) from each sample was reverse-transcribed in a 10 μL reaction using an AMV RNA PCR Kit 3.0 (Takara). The sequences of the primers used are shown in Table 5. The 18S rRNA gene and *UBQ7* gene of cotton were used as internal control genes. qRT-PCR was performed using a SYBR Premix Ex Taq™ Kit (Takara) according to the manufacturer’s protocol. The selected genes were verified using a Bio-Rad iQ5 real-time PCR detection system with a cycling temperature of 57°C and with a single peak on the melting curve to ensure a single product. At least three replicates were tested per sample.

**Table 5:** primers for qRT-PCR

**Description**	**id**	**ID**	**F**	**R**
Celullose synthase-like D protein	unigene5503_kv-1	1	CAATGCGTGAGTCCAAGT	AAAGCCATTAGCACCTGA
Xyloglucan endotraglucosylase/hydrolase	unigene68082_kv-1	2	GCAACTGAAGATGGCAAAT	GACGAGTAAGCCGAGCAA
Terpene synthase 3	unigene52341_kv-1	3	ATTGGAGTTTGCTAGGGATC	AGGAAATGGGCTTTGTGA
Oxidoreductase	unigene5647_kv-1	4	TGAAGTTGGGTGGGAGAA	CAGGCTGTGGACATTTGG
Early-responsive to dehydration protein-related	unigene23926_kv-1	5	TTGATGCTGAAACCGCTTAT	ATTGCCTCTTCCCTGTCCT
Early-responsive to dehydration protein-related	unigene23927_kv-1	6	TTATGCTACCGTGACACCT	CAACAGCCAGAGTAATGAGA
Xyloglucan 6-xylosyltransferase	unigene67909_kv-1	7	CGAAAGGGAAGGTTAGAG	TAAACTATGGCGGACTGA
Cytochrome P450	unigene25666_kv-1	8	TTGGTGGAGATGAAATGTG	TGGACTAGAATGGGTAAGC
GSDL-motif lipase	unigene5707_kv-1	9	TGCCATACTTGAGCCCTGAC	TAGCCGCTGTGCCTGTTG
AP2/ERF domain-containing transcription factor	unigene11425_kv-1	10	GGGCAGCCGAGATAAGAG	TTGGGTCAGTAGATGTAGGAAT
AP2/ERF domain-containing transcription factor	unigene13946_kv-1	11	TCCGTTGCTTCGTATCCT	AACCCTTCATCTTTGACCAC
Symbiotic ammonium transporter	unigene64452_kv-1	12	GAAAGCCAATCTAACAATC	TCAACAAAGCCAGTCGTA
Protein TIFY 9	unigene56521_kv-1	13	AGGGCTACATCCGAGAAT	AGGAGGACTTGCCACTTT
MYB transcription factor	unigene54286_kv-1	14	TCAGTCGAGGAAGTAAGAA	GTATAGGGATTTGACCAGA
Mitogen-activated protein kinase kinase kinase 17	unigene9429_kv-1	15	AGTGTCGTCGTCAGTTTCC	GGTATTTCAGGCACATCAG
F-box family protein-like	unigene4485_kv-1	16	GCTTTAGCTTCGGCTGTC	CCAAATCGCTTTCACTCA
Calmodulin-binding-like protein	unigene51427_kv-1	17	TTGGTTGCTCGTGATGGA	TTGCCCGTATGAGTTGTC
NAC domain protein	unigene25035_kv-1	18	AGTGATCGGGATGAAGAAA	CGGCTGGATTATAGTGGTC
Amino acid transporter	unigene60928_kv-1	19	TGAGTTTGGAGGGCTTAC	GCATTGAACTGTAGAGGGTA
Anion exchanger family protein	unigene12430_kv-1	20	TACTTCCTGGTATGTTTCG	ATTCTCCTCTGTGCGTTG
18 s	L24145	21	TCGTAGTTGGACTTAGGGTGGG	CAAATGCTTTCGCAGTTGTTCG
UBQ7	DQ116441	22	GAAGGCATTCCACCTGACCAAC	CTTGACCTTCTTCTTCTTGTGCTTG

## Competing interests

The authors declare that they have no competing financial interests.

## Authors’ contributions

YFC conceived the study. HZJ, QS, XYZ , XHH, YZS,YLY and XMD participated in experiment materials preparation. XYZ, QS, WNW participated in RNA extraction. QS analyzed data and performed qRT-PCR. YFC, QS wrote the paper. All authors read and approved the final manuscript.

## Supplementary Material

Additional file 1 **The distribution of different expression genes in MapMan software function annotation.** Note: column 1 showed BinCode ID. Column 2 meant the bin name of corresponding Bincode id or metabolic pathways name. Click here for file
